# Genome-Wide Identification and Expressional Profiling of the *Metal Tolerance Protein* Gene Family in *Brassica napus*

**DOI:** 10.3390/genes13050761

**Published:** 2022-04-26

**Authors:** Tao Xie, Wenjing Yang, Xin Chen, Hao Rong, Youping Wang, Jinjin Jiang

**Affiliations:** 1 Jiangsu Provincial Key Laboratory of Crop Genetics and Physiology, Yangzhou University, Yangzhou 225009, China; 007521@yzu.edu.cn (T.X.); wjyang1205@126.com (W.Y.); 18705275569@163.com (X.C.); wangyp@yzu.edu.cn (Y.W.); 2 Joint International Research Laboratory of Agriculture and Agri-Product Safety, The Ministry of Education of China, Yangzhou 225009, China; 3 School of Biological and Food Engineering, Suzhou University, Suzhou 234000, China; d160114@yzu.edu.cn

**Keywords:** *Brassica napus*, *Metal Tolerance Protein* gene, heavy metal, comparative studies, expression pattern

## Abstract

The *Cation Diffusion Facilitator* (*CDF*) family, also named *Metal Tolerance Protein* (*MTP*), is one of the gene families involved in heavy metal transport in plants. However, a comprehensive study of MTPs in *Brassica napus* has not been reported yet. In the present study, we identified 33 *BnMTP* genes from the rapeseed genome using bioinformatic analyses. Subsequently, we analyzed the phylogenetic relationship, gene structure, chromosome distribution, conserved domains, and motifs of the *BnMTP* gene family. The 33 BnMTPs were phylogenetically divided into three major clusters (Zn-CDFs, Fe/Zn-CDFs, and Mn-CDFs) and seven groups (group 1, 5, 6, 7, 8, 9, and 12). The structural characteristics of the BnMTP members were similar in the same group, but different among groups. Evolutionary analysis indicated that the *BnMTP* gene family mainly expanded through whole-genome duplication (WGD) and segmental duplication events. Moreover, the prediction of cis-acting elements and microRNA target sites suggested that *BnMTPs* might be involved in plant growth, development, and stress responses. In addition, we found the expression of 24 *BnMTPs* in rapeseed leaves or roots could respond to heavy metal ion treatments. These results provided an important basis for clarifying the biological functions of *BnMTPs*, especially in heavy metal detoxification, and will be helpful in the phytoremediation of heavy metal pollution in soil.

## 1. Introduction

With the development of industrial and agricultural modernization, and the urbanization of human society, the heavy metal contamination of agricultural land and water has become one of the main restrictive factors affecting plant growth and crop yield [1,2,3]. Metal cations, such as Zn, Mn, and Cu, are necessary trace elements that could serve as catalytic or structural cofactors of enzymes and regulatory factors in regulating the various life activities of plants [4,5,6]. However, excessive trace elements are toxic to plants [7,8]. Therefore, their concentration in plant cells must be strictly controlled to achieve optimal growth. On the other hand, some unnecessary heavy metals, such as Hg, Cd, Cr, and Pb, are toxic to plant growth and development even in small doses [9]. In addition, these toxic heavy metals could be easily transported and accumulated in different organs of crops (e.g., root, stem, leaf, and seed), thus causing severe damage to animal and human health [10,11]. Under long-term heavy metal stress, plants have gradually formed two adaptation mechanisms to heavy metal toxicity. The first is to restrict the metal influx across the membrane, increase the metal binding to the cell wall, and stimulate the efflux pumping of metals from cytosol, to maintain the low metal concentration in the cytoplasm [12]. The second is to chelate the absorbed heavy metals with organic molecules and transform them into non-toxic forms (e.g., phytochelatin or metallothionein), then transport and accumulate the metal complex into the vacuole for metal detoxification [12,13,14]. Hitherto, many metal transporters have been reported in plants (e.g., *Arabidopsis thaliana*, *Oryza sativa*) that play key roles in heavy metal detoxification [11,15,16,17].

Cation Diffusion Facilitator (CDF), also named Metal Tolerance Protein (MTP), is a conserved and ancient protein family, which is important in transporting heavy metal ions from the cytoplasm into specific organelles [18]. Since it was first described in *Cupriavidus metallidurans* [19], MTPs have been found in Archaea, Eubacteria, and Eukaryotes [1,18,20]. According to the evolutionary relationship of MTP family members and their substrate specificity, MTPs are classified into three major clusters: Zn-CDFs, Zn/Fe-CDFs, and Mn-CDFs [18]. These family members are usually characterized with three typical features: an N-terminal specific sequence, a C-terminal cation efflux domain (PF01545), and six transmembrane domains (TMDs) [18,21,22].

To date, many plant CDF/MTP transporters have been reported. In *A. thaliana*, 12 *MTP* genes were identified and divided into seven groups (group 1, 5, 6, 7, 8, 9, and 12) through phylogenetic analysis [1]. Among them, group 1 (MTP1-4), 5 (MTP5) and 12 (MTP12) belong to the Zn-CDFs, group 6 (MTP6) and 7 (MTP7) belong to the Zn/Fe-CDFs, while group 8 (MTP8) and group 9 (MTP9-11) form Mn-CDFs [1,23]. Most Zn-CDF members are involved in the transport and distribution of Zn, but in different molecular mechanisms. AtMTP1 is located in the vacuolar membrane of root and leaf cells and is involved in maintaining zinc homeostasis through transporting excess zinc from the cytoplasm to the vacuoles [7,24]. The expression of *AtMTP3* in root epidermal and cortical cells could be significantly induced under a non-toxic concentration of Zn [8]. AtMTP3 protects cells from Zn toxicity by metal partitioning, particularly under high Zn influx in *Arabidopsis* root [8,25]. AtMTP12 forms a complex with AtMTP5 to transport zinc into the Golgi apparatus, thus maintaining the intracellular zinc homeostasis [26]. In *Cucumis sativus*, the heterodimeric complex CsMTP12–CsMTP5 was also functioned in transporting Zn into Golgi [27]. Besides Zn transportation, Zn-CDF members are also involved in delivering other divalent cations. In *O. sativa*, *MTP1* could be induced by Cd treatment and is involved in transporting Zn, Cd, Co, Fe, and Ni [5,28,29]. The ectopic expression of *OsMTP1* in yeast increases the tolerance to Zn, Cd, and Ni [28]. The expression of wheat *MTP1* in yeast also enhances Zn and Co resistance [30]. Unlike the Zn-MTPs, only a few Mn-CDFs are functionally reported. AtMTP8/OsMTP8, AtMTP11/OsMTP11, and OsMTP9/CsMTP9 have been proven to have functions in Mn transport [31,32,33,34,35,36]. Hitherto, the function and metal specificity of Fe/Zn-CDFs (MTP6 and MTP7) have not been reported yet. Besides metal transport, plant MTPs may also participate in other physiological processes. The *Arabidopsis* IAA-Ala-resistant mutant *iar1* is resistant to a variety of IAA-amino acid conjugates, and *MTP5* mutation restores IAA-conjugate sensitivity to *iar1* [37]. In rice, *MTP11* mutation decreases grain yield and fertility, but does not affect the tolerance and accumulation of Mn [38].

Phytoremediation is an economical and environmentally friendly soil cleaning technology [39]. *Brassica napus* is an ideal crop in phytoremediation for its high capacity of heavy metal enrichment [39,40,41]. As reported, *B. napus* could produce safe oil even when grown on soil with severe contamination of different heavy metals, since the heavy metals are mainly retained in the residues after oil extraction [40,42]. In hyperaccumulator plants, the constitutive overexpression of transporter genes, including *MTPs*, are important to the absorption, transport, and finally isolation of different heavy metals inside cells of specific organs [43]. Therefore, a comprehensive study of the *MTP* gene family is of great significance to improve plant resistance to heavy metals and soil contamination. Based on the genome data of various plant species, MTPs have been widely identified, including turnip, rice, tobacco, *Vitis vinifera*, *Populus trichocarpa,* and *Camellia sinensis* [23,44,45,46,47,48]. However, the whole genome analysis and functional study of the *MTP* gene family in *B. napus* have not been reported. In this study, we comprehensively analyzed the evolution, structure, conserved motif, chromosome localization, subcellular localization, and expression pattern of 33 *MTPs* in *B. napus*. This study is important for the functional research of *BnMTPs* in the future, and provides a new idea for creating rapeseed germplasm with heavy metal resistance or high soil remediation abilities.

## 2. Materials and Methods

### 2.1. Identification of MTP Genes in B. napus Genome

Twelve *Arabidopsis MTP* sequences were downloaded from the TAIR (https://www.arabidopsis.org/, accessed on 5 November 2021). The genome and protein sequences of *B. napus* (Darmor-*bzh*) were obtained from the Genoscope database (http://www.genoscope.cns.fr/brassicanapus/, accessed on 5 November 2021). To identify the *MTP* genes in *B. napus*, the conserved domain of MTPs (Cation_efflux, Pfam number: PF01545) acquired from Pfam (http://pfam.xfam.org/, accessed on 5 November 2021) was used as a query to blast against the peptide sequences of *B. napus* with an e-value less than 1 × 10^−10^ [49]. Then, the MTP domain in the predicted BnMTPs was screened again with HMMER (https://www.ebi.ac.uk/Tools/hmmer/, accessed on 7 November 2021) and InterProScan (https://www.ebi.ac.uk/interpro/search/sequence/, accessed on 7 November 2021) [50,51], and the candidates containing the typical cation efflux domain were recognized as BnMTPs. The *MTPs* in *B. rapa* and *B. oleracea* were obtained as mentioned above. All of the candidate *MTPs* were renamed according to the homology to *AtMTPs*. In addition, *BnMTPs* from the pangenome of rapeseed were retrieved from the BnPIR database (http://cbi.hzau.edu.cn/bnapus/index.php, accessed on 5 November 2021) [52].

### 2.2. Phylogenetic Analysis and Characterization of BnMTPs

The multiple sequence alignment of MTPs was performed using ClustalX (http://www.clustal.org/clustal2/, accessed on 10 November 2021). The phylogenetic tree was constructed using MEGA v.6.0 with the neighbor-joining (NJ) method using the p-distance and pairwise deletion option, and with a bootstrap analysis of 1000 replicates.

The number of amino acids, molecular weight (MW), and theoretical isoelectric point (pI) of the BnMTPs were calculated using the protein isoelectric point calculator (http://isoelectric.org/, accessed on 10 November 2021). The subcellular localization of BnMTPs were predicted with ProtComp v.9.0 in Softberry (http://linux1.softberry.com/, accessed on 10 November 2021). The TMHMM Server v.2.0 was used to predict the transmembrane region of BnMTPs. The exon–intron structures of *BnMTPs* were extracted from the genome annotation file of *B. napus*. The conserved motif and domain in BnMTPs were predicted by MEME (http://meme-suite.org/tools/meme, accessed on 10 November 2021) and Pfam, respectively. The gene structure, conserved motifs, and domains were visualized by TBtools [53].

### 2.3. Gene Location and Duplication Analysis of BnMTPs

MG2C (http://mg2c.iask.in/mg2c_v2.0/, accessed on 12 November 2021) was used to generate the location image of *MTPs* on the chromosomes of the *B. napus* genome. MCScanX was used to analyze the collinear blocks and gene replication events among *B. napus*, *A. thaliana*, *B. rapa*, and *B. oleracea* with default parameter settings. The collinear maps among *B. napus* and other species were visualized by TBtools. Nonsynonymous (*Ka*) and synonymous (*Ks*) values, and the *Ka/Ks* ratios were calculated by ParaAT 2.0 (https://bigd.big.ac.cn/tools/paraat, accessed on 12 November 2021) and KaKs_Calculator 2.0 [54].

### 2.4. Cis-Acting Regulatory Element and MicroRNA Target Site Analysis of BnMTPs

The 2 kb upstream sequences of *BnMTPs* were obtained using TBtools, and *cis*-acting elements were analyzed with the PlantCare database (http://bioinformatics.psb.ugent.be/webtools/plantcare/html/, accessed on 13 November 2021). The miRNA target sites of *BnMTPs* were predicted using psRNATarget server (https://www.zhaolab.org/psRNATarget/, accessed on 13 November 2021) [55].

### 2.5. Temporospatial Expression Analysis of BnMTPs Based on RNA-seq Data

To examine the tissue expression pattern of the *BnMTP* genes, three replicates of 15 samples representing the major developmental tissues and organs of *B. napus* were used for RNA-seq analysis, including leaf, cotyledon, hypocotyl, root, shoot apical meristem (SAM), stem, bud, flower, endosperm, silique, and five seed samples at 3, 4, 5, 6 weeks after flowering (WAF) and mature stage. Using the RNA-seq data, a heat map of *BnMTPs* in different developmental stages was generated based on the log_10_ transformed values of fragments per kilobase of transcript per million fragments mapped (FPKM) values. If FPKM = 0, log_10_FPKM was artificially defined as −3.

### 2.6. Plant Materials, Growth Conditions and Treatments

To acknowledge the expression level of *BnMTPs* under different abiotic stresses, the seeds of rapeseed were germinated and transferred to 1/2 MS medium containing 15% PEG, 150 mM NaCl, or 150 mM mannitol according to previous studies with several modifications [56,57]. The seedlings were grown in a climate chamber under a photoperiod of 16 h light/8 h dark, at 22 °C for 14 days. For cold treatment, the seedlings were grown at 22 °C for 12 days and 4 °C for 2 days. Three biological replicates of 10 seedlings were pooled for each treatment. In order to examine the expression pattern of *BnMTPs* under hormone stimuli, we conducted hormone treatments as described by previous studies with several modifications [56,58,59,60]. Briefly, five-week-old *B. napus* seedlings cultured in nutrition soil in a greenhouse were sprayed with different hormone solutions, including 100 μM abscisic acid (ABA), 500 μM gibberellin (GA), 50 μM indoleacetic acid (IAA), 100 μM kinetin (KT), and 10 μM strigolactone (SL). Leaf samples were collected at 0 h, 1 h, and 3 h after treatment. Three biological replicates of six leaves from three seedlings were pooled for each treatment. The abovementioned samples were ground into powder and stored at −80 °C for RNA-seq. The heatmap of *BnMTP* expression under abiotic stresses and hormone treatments was generated based on the log_2_ transformed FPKM ratios, using the control group as CK. If the FPKM values were lower than 1, it was considered that there was no significant difference between the CK and test samples, and the log_2_ transformed ratio was defined as 0. The *BnMTPs* were clustered according to hierarchical clustering.

To investigate the response of *BnMTPs* under different heavy metal treatments and selenium, rapeseed seeds were germinated on wet filter papers for two days and moved into an aperture disk containing vermiculite. The seedlings were watered with Hoagland nutrient solution, and grown with a 16 h light/8 h dark photocycle. The three-week-old plants were irrigated with Hoagland nutrient solution containing different metal ions, including 100 μM CuCl_2_, 100 μM ZnSO_4_, 100 μM MnCl_2_, 180 uM HgCl_2_, 100 μM Pb(NO_3_)_2_, 400 μM K_2_Cr_2_O_7_ and 1 μM Na_2_SeO_3_. After 8 days of treatment, the roots and leaves from five seedlings were pooled separately for RNA isolation and qRT-PCR analysis. The plants irrigated with Hoagland nutrient solution were used as a control.

### 2.7. RNA Isolation and qRT-PCR Analysis

The total RNA was isolated using RNA isolator Total RNA Extraction Reagent (Vazyme, Nanjing, China) according to the manufacturer’s instructions. A total of 3 μg of RNA was reverse transcribed using the HiScript III RT SuperMix for qPCR (Vazyme, Nanjing, China) to generate the cDNA. PowerUp SYBR Green Master Mixes (Thermo, Waltham, MA, USA) and a StepOnePlus Real-Time PCR System (Thermo, Waltham, MA, USA) were used to perform qRT-PCR analysis. *B. napus Actin-7* (NC_027775.2) was used as an internal control. The reactions were carried out according to the following program: 95 °C for 15 min, 40 cycles followed by 95 °C for 15 s, 60 °C for 1 min. The 2^−^^△△Ct^ method was used to calculate the relative expression level of *BnMTPs*. Each experiment was technically repeated three times. All of the primers were synthesized by TSINKE Biotech and are listed in Table S1.

### 2.8. Statistical Analysis

Statistical analysis was performed using SPSS 19.0. One-way ANOVA or independent-samples *t*-test were used to analyze significant differences among multiple samples or between each pair of samples at a 0.05 level, respectively.

## 3. Results

### 3.1. Identification and Phylogenetic Analysis of BnMTP Proteins

**Figure 1:**
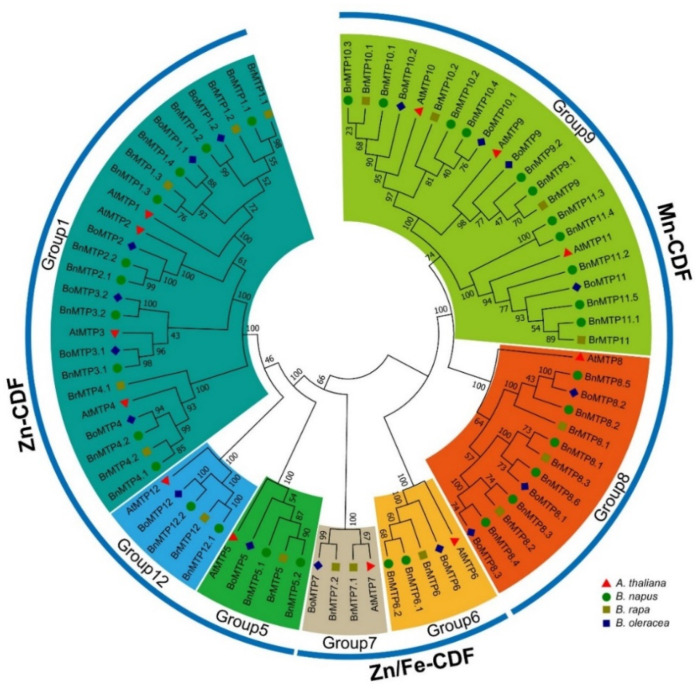
Phylogenetic relationships of MTP proteins in *A. thaliana*, *B. rapa*, *B. oleracea*, and *B. napus* using MEGA 6.0 with the neighbor-joining (NJ) method. The numbers at the nodes represent the reliability percentage of the bootstrap values based on 1000 replications. The red triangle, green circle, brown square, and blue diamond represent MTPs from *A. thaliana*, *B. rapa*, *B. oleracea*, and *B. napus,* respectively.

A total of 33 BnMTPs, 17 BrMTPs, and 17 BoMTPs were identified in *B. napus*, *B. rapa*, and *B. oleracea*, respectively. The BnMTPs were named as BnMTP1.1 to BnMTP12.2 according to the similarity and phylogenetic relationship to AtMTPs (Table S2). For each *AtMTP* gene, at least two *BnMTP* orthologs were identified on the *B. napus* genome except for *AtMTP7*. A phylogenetic tree of 79 MTPs from *A. thaliana*, *B. rapa*, *B. oleracea*, and *B. napus* classified the MTPs into three major clusters: Zn-CDFs, Zn/Fe-CDFs, and Mn-CDFs, and the three clusters were further divided into seven groups (i.e., group 1, 5, 6, 7, 8, 9, and 12) (Figure 1). Group 9 was the largest group, containing 11 BnMTPs, but no BnMTP was included in group 7. There were ten BnMTPs in group 7 and six BnMTPs in group 8, and only two BnMTPs were classified in each of the group 5, 6, and 12.

The characteristics of BnMTP proteins are listed in Table S2, including the gene identifier (gene ID), CDS length, protein size, chromosome location, pI, MW, and TMD number. The length of the *BnMTPs* ranged from 456 bp (*BnMTP2.1*) to 2310 bp (*BnMTP12.1*), with encoding proteins ranging from 151 to 769 amino acids. The MW of the BnMTPs ranged from 16.95 kDa (BnMTP2.1) to 86.67 kDa (BnMTP12.1), and most of them were within 40 to 50 kDa. The pI of most BnMTPs was lower than 7, except for BnMTP6.1, indicating that most *BnMTPs* may function in an acidic environment. All of the 33 BnMTPs were predicted with localization in the vacuole, and four of them were also located in the cell membrane. We found 24 BnMTPs containing 4–6 TMDs, whereas other BnMTPs harbored 3 (BnMTP2.1, BnMTP11.3, and BnMTP11.4), 7 (BnMTP5.1 and BnMTP10.4), and 16 (BnMTP12.1 and BnMTP12.2) TMDs. However, no TMD was identified in BnMTP6.1 or BnMTP6.2.

### 3.2. Structural and Chromosomal Localization Analysis of BnMTP Genes

**Figure 2:**
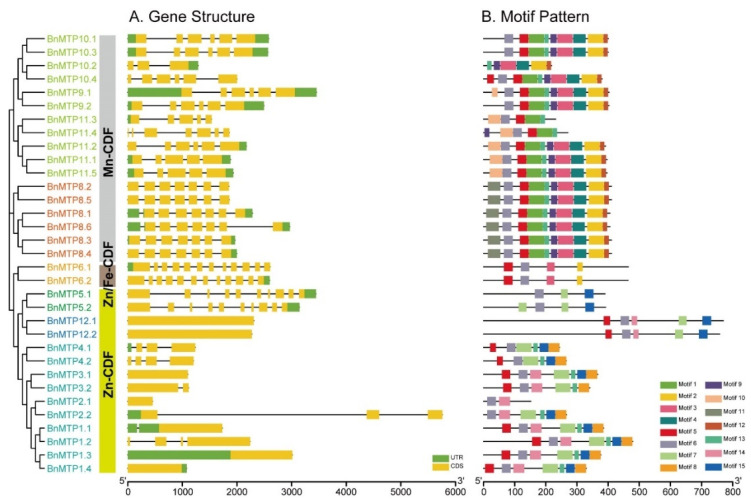
The gene structure and motif pattern of the *MTP* gene family in *B*. *napus*. (**A**) The exon–intron structure of *BnMTP* genes. Green boxes indicate 5′- and 3′-untranslated regions (UTR), yellow boxes indicate exons, and black lines indicate introns. (**B**) Conserved motifs of BnMTP proteins. Motif 1–15 identified by MEME analysis are displayed in different colored boxes.

The genome annotation file from the Genoscope database of *B. napus* was used to analyze the exon–intron structure of the *BnMTPs*. The closely related *BnMTP* members showed similar exon–intron structure and gene length, which was consistent with the phylogenetic relationship mentioned above. We found the number of introns in *BnMTPs* varied dramatically, ranging from 0 to 11, but seven *BnMTPs* in the Zn-CDF clade had no intron (Figure 2A). Other *Zn-CDFs* in *B. napus* contained one to three introns, except for *BnMTP5.1* and *BnMTP5.2* in group 5, which contained nine introns. The two Zn/Fe-CDFs in *B. napus* were identified with 11 introns. The intron number of *Mn-CDFs* in *B. napus* ranged from two to six, of which members in group 8 had six introns, and *BnMTPs* in group 9 contained four to six introns (except for *BnMTP10.2*).

**Figure 3:**
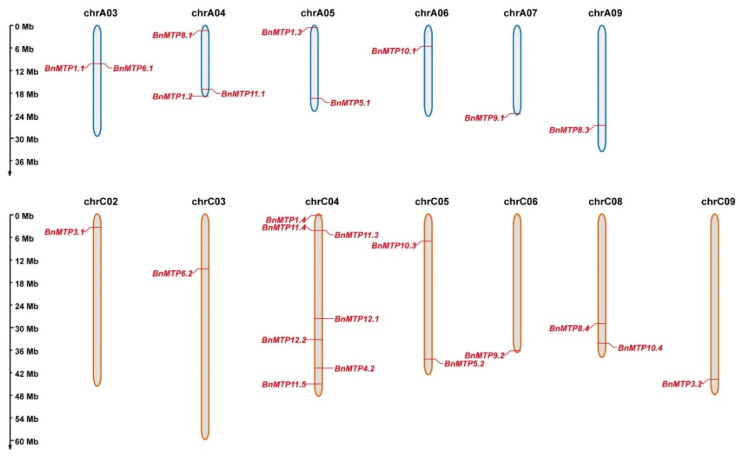
Chromosomal location of *BnMTP* genes. The unmapped eight *BnMTPs* are located on unassembled scaffolds. The scale of the chromosomes is in megabases (Mb).

Among the 33 *BnMTPs* (15 on A-subgenome and 18 on C-subgenome), we found that 25 *BnMTPs* were unevenly distributed on 13 chromosomes of *B. napus*, and 8 *BnMTPs* were not assigned to specific chromosomes (Figure 3). Chromosome C04 contained the largest number of *BnMTPs* (seven genes), chromosome A04 contained three *BnMTPs*, chromosomes A03, A05, C05, and C08 contained two *BnMTP* genes. While chromosomes A06, A07, A09, C02, C03, C06, and C09 were assigned with only one *BnMTP* gene, no *BnMTP* gene was located on chromosomes A01, A02, A08, A10, C01, or C07. In addition, we found no significant correlation between the chromosome length and number of *BnMTPs*.

### 3.3. Conserved Domain and Motif of BnMTP Proteins

**Figure 4:**
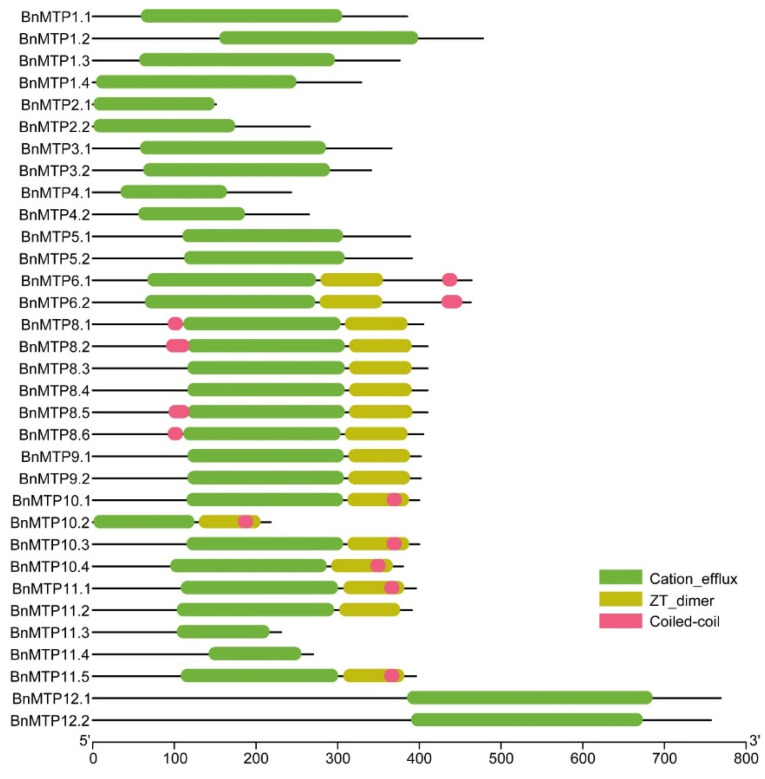
Distribution of the conserved domain in BnMTP proteins. Green boxes indicated the cation_efflux domain; yellow boxes indicated ZT_dimers, and pink boxes indicated a coiled-coil structure.

The multiple sequence alignment of AtMTPs and BnMTPs revealed that all three clusters contained a conserved CDF signature (44 amino acids) at the N terminus. Similar to previous studies, two conserved HxxxD (x = any amino acid) residues were identified in the Zn-CDFs of *B. napus*, except for BnMTP2.1 and BnMTP2.2. One conserved HxxxD residue was contained in Zn/Fe-CDFs, and two DxxxD residues were found in the Mn-CDFs of *B. napus*. Additionally, the BnMTPs in group 1 and 12 contained a His-rich region (Figure S1). We used HMMER to analyze the domains of BnMTPs, and found that all of the members contained the cation_efflux domain. The BnMTPs in group 6, 8, and 9 also contained a dimerization domain of Zinc Transporter (ZT_dimer), except for BnMTP11.3 and BnMTP11.4. In addition, a coiled-coil structure was identified in the BnMTPs of group 6, 8, and 9 (except for BnMTP8.3, BnMTP8.4, BnMTP9.1, BnMTP9.2, BnMTP11.2, BnMTP11.3, and BnMTP11.4) (Figure 4). The motif analysis revealed 15 conserved motifs in *BnMTPs*, with a length ranging from 6 to 50 amino acids (Figure 2B, Table S3). The motif sequences were submitted to Pfam and five conserved motifs might encode functional domains. Motif 1, 3, and 7 encoded Cation_efflux, motif 2 encoded ZT_dimer, while motif 10 might encode vacuolar ATP synthase subunit S1 (ATP-synt_S). Motif 6 was the most conserved motif in the BnMTPs, except for BnMTP10.2. Motif 5 and 13 existed in 28 and 26 BnMTP proteins, respectively. In general, BnMTPs in the same cluster or group were identified with a similar type and distribution of conserved motifs. For instance, most Mn-CDFs contained motif 1, 4, 9, and 12, except for BnMTP10.2, BnMTP11.3, and BnMTP11.4. Motif 2 and 3 were only present in Mn-CDFs and Zn/Fe-CDFs. Motif 10 and 11 were specific to the Mn-CDFs in group 9 and 8, respectively. Nearly all of the Zn-CDFs in *B. napus* contained motif 7 and 15, except for BnMTP2.1. Motif 8 only existed in Zn-CDFs in group 1, and motif 14 was specific to Zn-CDFs in group 1 and 12. Among the three clusters, Mn-CDFs contained the largest number of motifs (group 8 contained 10 motifs, and group 9 contained 9–10 motifs), except for BnMTP10.2, BnMTP11.3, and BnMTP11.4 that contained 5–6 motifs. In addition, the number, type, structure, and position of the motifs in Mn-CDFs were more similar than in the other two clusters. These unique and different conserved motif structures may lead to functional differences in BnMTP proteins.

### 3.4. Synteny Analysis of MTP Genes

**Figure 5:**
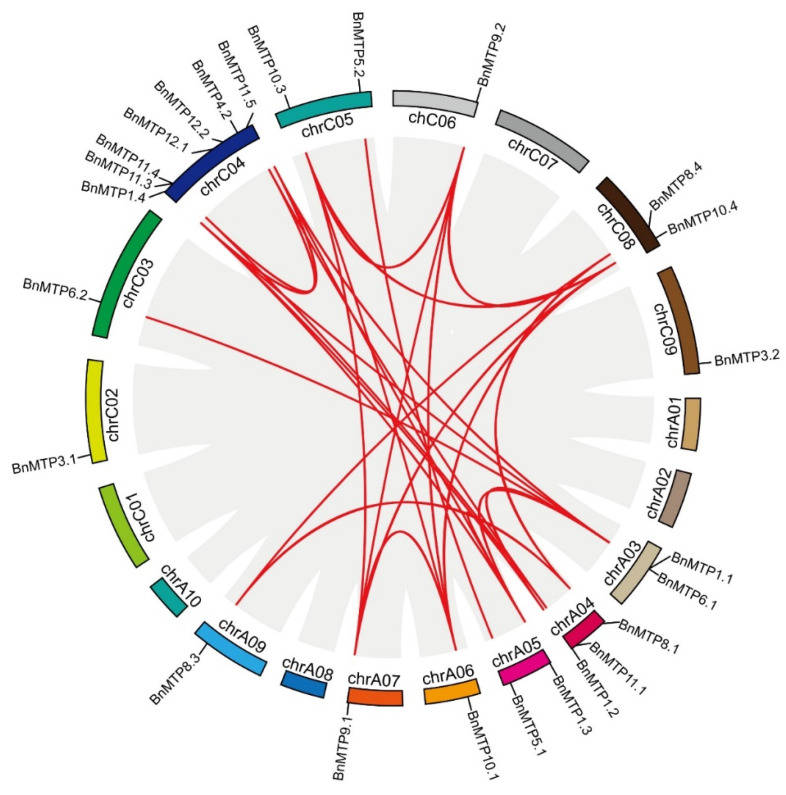
Chromosomal distribution and syntenic relationships of *MTP* genes in *B. napus* genome. Gray lines indicate all synteny blocks in the *B. napus* genome, and the red lines indicate duplicated *BnMTP* gene pairs.

The BLASTP and MCScanX were used to identify the homologous genes and the duplication events in the *BnMTP* gene family. A total of 29 pairs of *BnMTP* paralogs were found in *B. napus* (Figure 5, Table S4). We found that all of the *BnMTPs* resulted from duplication events, of which 22 *BnMTPs* were derived from whole-genome duplication (WGD) or segmental duplication, 9 *BnMTPs* from dispersed duplication, and 2 *BnMTPs* from proximal duplication (Table S5). These results suggest that gene duplication events, especially WGD and segmental duplication, played essential roles in the generation and evolution of the *BnMTP* gene family.

**Figure 6:**
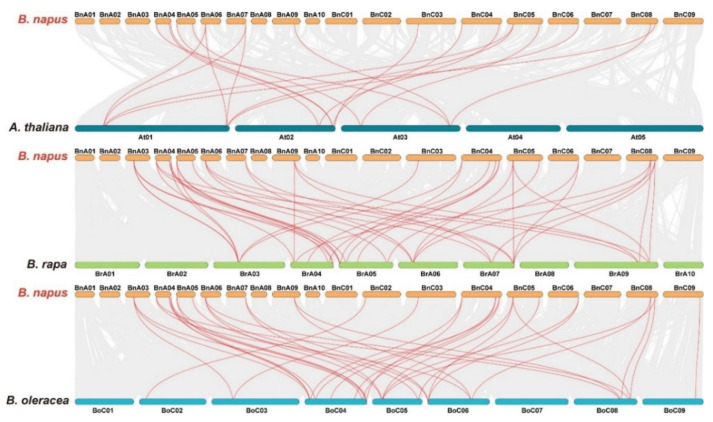
Synteny analysis of *MTP* genes in *B. napus* and three ancestral plant species. Gray lines in the background indicate the collinear blocks within *B. napus* and other plant genomes, while the red lines highlight the syntenic *MTP* gene pairs.

**Figure 7:**
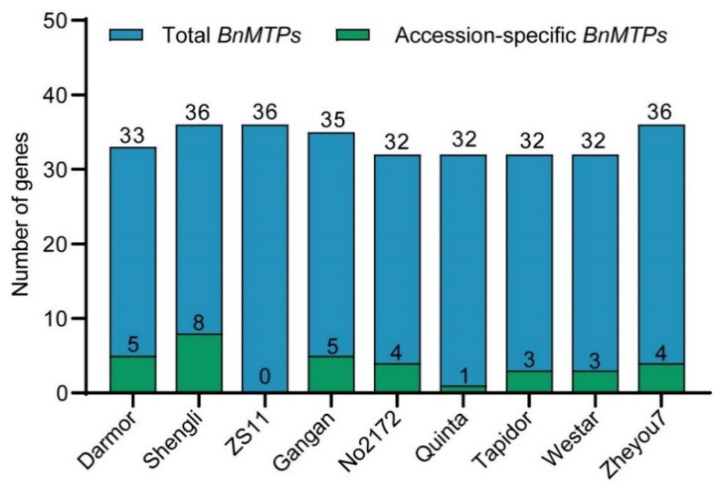
The number of *BnMTP* genes in different rapeseed accessions. The blue column represents the total *BnMTPs* and the green column represents the accession-specific *BnMTPs*.

To further understand the evolutionary relationship of *MTP* family members, collinearity analyses were conducted between *B. napus* and *A. thaliana*, *B. napus* and *B. rapa*, and *B. napus* and *B. oleracea*. A total of 22 *BnMTPs* (66.67%) were identified with collinearity relationship to *MTPs* in other *Brassicaceae* species, of which 19, 42, and 38 orthologous gene pairs were identified from *B. napus–A. thaliana*, *B. napus–B. rapa*, and *B. napus–B. oleracea*, respectively (Figure 6). Moreover, we screened the *BnMTPs* in the rapeseed pangenome and found 36 *BnMTPs* in ZS11, Shengli, and Zheyou7, 35 *MTPs* in Gangan, and 32 genes in No2172, Quinta, Tapidor, and Westar, which were similar to the *MTPs* in Damor-*bzh* (Figure 7). We found five, eight, five, four, one, three, three, and four accession-specific *MTPs* in Damor-*bzh*, Shengli, Gangan, No2172, Quinta, Tapidor, Westar, and Zheyou7, respectively. In general, these results show that gene duplication events were the main forces promoting the expansion of the *MTP* gene family in *B. napus*, and the *MTPs* were conserved in different *B. napus* accessions.

To characterize the selective pressure of duplicated *MTP* genes in the course of evolution, ParaAT2.0 and KaKs Calculator 2.0 were used to calculate nonsynonymous (Ka) and synonymous (Ks) values, and the *Ka/Ks* ratio of orthologous gene pairs among *B. napus*, *B. rapa*, *B. oleracea*, and *A. thaliana* (Table S4). The *Ka/Ks* ratios of all *MTP* gene pairs were less than 0.5, indicating that the *MTP* family in *B. napus* and its ancestral species experienced intense purifying selection during the evolutionary process. The divergence time of homologous gene pairs between *B. napus* vs. *B. napus*, *B. napus* vs. *B. rapa*, *B. napus* vs. *B. oleracea*, and *B. napus* vs. *A. thalian* respectively ranged from 0.356 to 0.020, 0.354 to 0.011, 0.343 to 0.003, and 0.318 to 0.116 million years ago, indicating that the divergence of *MTP* gene pairs between *B. napus* and *A. thaliana* was earlier.

### 3.5. Cis-acting Elements in the Promoter Regions of BnMTPs

**Figure 8:**
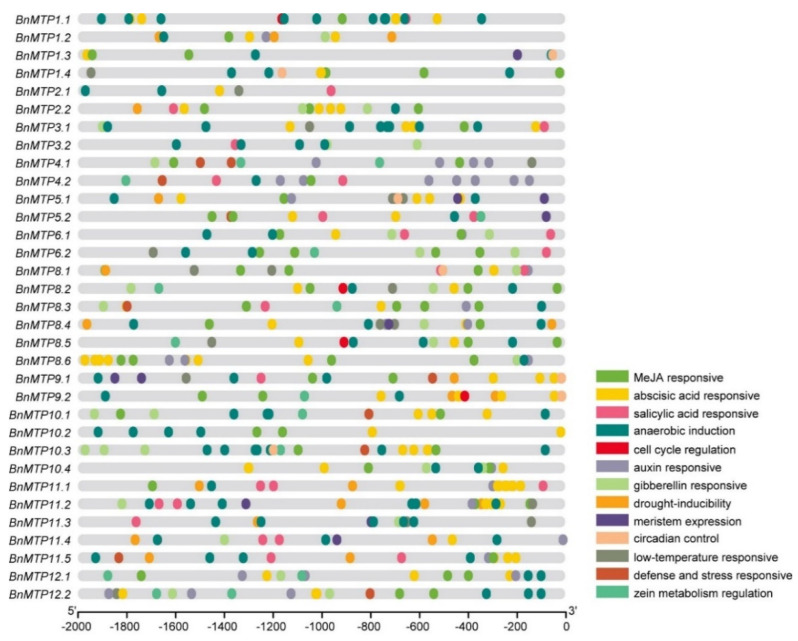
*Cis*-acting regulatory elements in the promoter region of *BnMTP* genes. The presence of different *cis*-acting elements was determined by the PlantCARE software. Different colored ovals represent different type of *cis*-elements.

The *cis*-acting element is a non-coding DNA region upstream of the gene coding region, which regulates the transcription of adjacent genes through the combination of some regulatory molecules. Using PlantCARE, a total of 2702 annotated *cis*-acting elements were identified in the promoter region of the *BnMTPs*, which were classified into 10 types, including gene transcription (1781 elements), light responsiveness (385 elements), phytohormone responsive (323 elements), abiotic stress responsiveness (151 elements), biotic stress responsiveness (3 elements), site-binding (20 elements), tissue expression (10 elements), secondary metabolism (18 elements), circadian control (7 elements), and cell cycle (4 elements) (Figure 8, Table S6). We found that gene transcription elements were the most abundant, including 1358 TATA-boxes and 423 CAAT-boxes. Light responsive elements were also common in the promoter regions of the *BnMTPs*, including 27 types of elements. The number of these common elements in *BnMTPs* ranged from 6 (*BnMTP 2.1* and *BnMTP 11.3*) to 23 (*BnMTP 11.1*), of which G-box, box 4, GT1 motif, and TCT motif were the most abundant elements. Moreover, 10 types of phytohormone responsive elements were identified in all *BnMTPs*, such as TGA-element, TGA-box, and AuxRR-core (auxin responsiveness), TATC-box, GARE-motif and P-box (GA responsiveness), TCA-element (salicylic acid responsiveness), CGTCA-motif and TGACG-motif (MeJA responsiveness), and ABRE (ABA responsiveness). We found the abscisic acid responsive elements were the most abundant, followed by the MeJA-responsive elements. Additionally, abiotic stress elements including ARE and GC-motif for anaerobic induction, LTR for low temperature responsiveness, MBS for drought inducibility, and TC-rich repeats for defense/stress responsiveness were also identified in the promoters of *BnMTP* genes. The *cis*-element analysis indicated that *BnMTPs* might be regulated by a variety of stimuli and be involved in plant growth and development, response to various stresses and hormones.

### 3.6. Potential miRNA Target Sites in BnMTP Genes

miRNAs are small, non-coding RNAs that regulate gene expression by interfering with mRNA transcription, translation, or epigenetic processes [61]. We analyzed the potential miRNA target sites in the *BnMTP* genes by psRNATarget (expectation score < 5.0), and found that 12 miRNAs targeting eight *BnMTPs* with the value of target accessibility–maximum energy to unpair the target site (UPE) of the miRNA/BnMTP varied from 4.093 to 23.236 (Table 1). *BnMTP8.1* had the target sites of *bna-miR1140* and *bna-miR156a/d/e/f*, *BnMTP8.3* and *BnMTP8.4* contained target sites for *bna-miR1140*, *BnMTP10.1* and *BnMTP10.3* were targeted by *bna-miR6031*, and *BnMTP10.4* were targeted by *bna-miR156b/c/g*. Both *BnMTP12.1* and *BnMTP12.2* were targeted by *bna-miR156a/d/e/f* and *bna-miR390a/b/c*. In addition, all of the identified miRNAs inhibited the *BnMTP* expression by cleaving the target sites.

### 3.7. Expression Profiles of BnMTP Genes in Different Tissues

**Figure 9:**
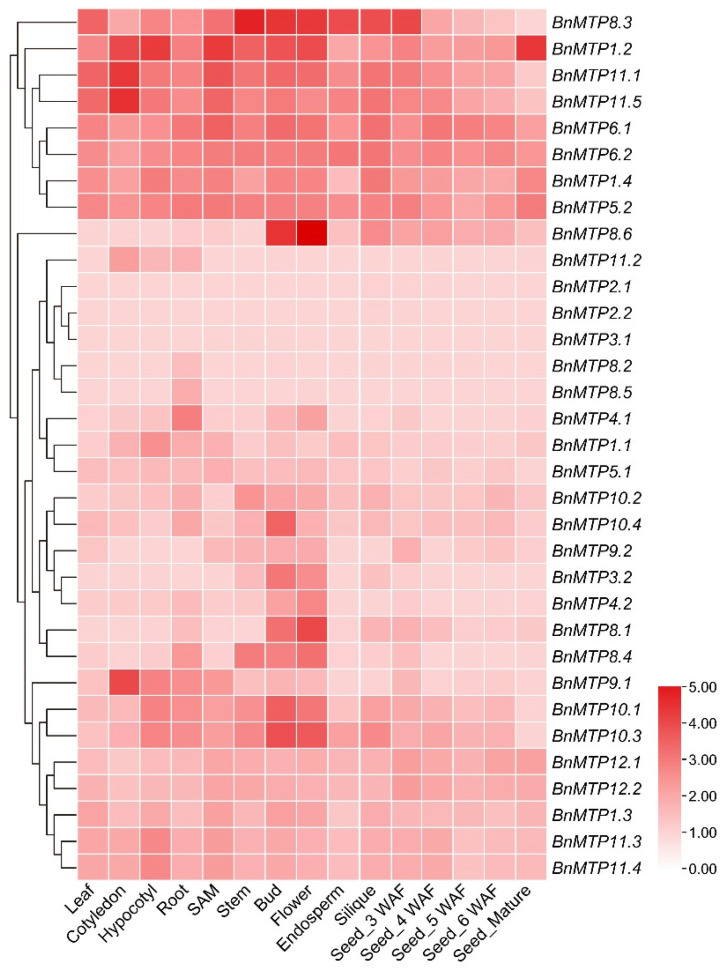
Temporospatial expression patterns of *BnMTP* genes. Normalized gene expression (FPKM + 1) is expressed in log_2_ ratio.

We found that the 33 *BnMTP* genes exhibited distinct temporal and spatial expression patterns in different tissues of *B. napus* (Figure 9, Table S7). Fifteen *BnMTP* genes were constitutively expressed during rapeseed development (log_2_(FPKM + 1) > 1), of which eight *BnMTPs* (*BnMTP1.2*, *BnMTP1.4*, *BnMTP5.2*, *BnMTP6.1*, *BnMTP6.2*, *BnMTP8.3*, *BnMTP11.1*, and *BnMTP11.5*) were highly expressed in most tissues, while the other genes (*BnMTP1.3*, *BnMTP10.1*, *BnMTP10.3*, *BnMTP11.3*, *BnMTP11.4*, *BnMTP12.1*, and *BnMTP12.2*) were identified with low expression. Moreover, some *BnMTP* genes were highly expressed in specific tissues. For instance, *BnMTP8.1*, *BnMTP8.6*, *BnMTP10.1*, *BnMTP10.3*, and *BnMTP10.4* were highly expressed in floral organs, and *BnMTP9.1*, *BnMTP11.1*, and *BnMTP11.5* were highly expressed in cotyledon. Four *BnMTP* genes (*BnMTP2.1*, *BnMTP2.2*, *BnMTP3.1*, and *BnMTP8.2*) were barely expressed in any of the tissues of *B. napus* (0 < log_2_(FPKM + 1) < 1).

### 3.8. Expression Pattern of BnMTPs under Abiotic Stress and Hormone Treatment

**Figure 10:**
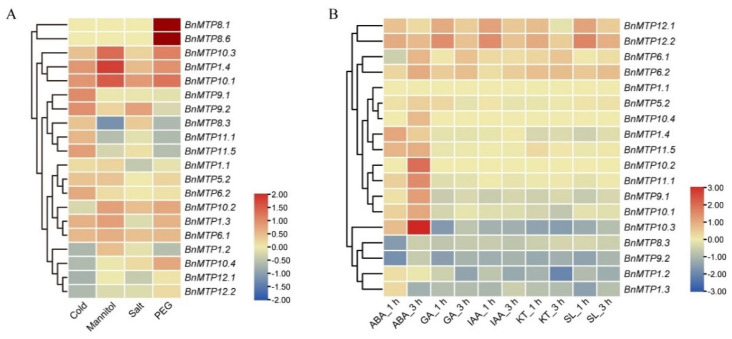
*BnMTP* genes in response to abiotic stresses and hormone treatments. (**A**) Gene expression under abiotic stress. (**B**) Gene expression under hormone treatment. Ratios of FPKM values under stress conditions to FPKM values under normal conditions were log_2_ transformed and used to represent the gene expression levels.

As indicated above, *cis*-acting elements related to abiotic stress and hormone response were abundant in the promoter of *BnMTP* genes. We examined the expression pattern of the *BnMTPs* under different abiotic stresses and hormone treatments using transcriptome sequencing (Figure 10, Table S8). Overall, some *BnMTPs* were significantly induced or repressed by abiotic stress treatments. For instance, *BnMTP1.4*, *BnMTP10.1*, and *BnMTP10.3* were simultaneously induced by cold, mannitol, and PEG treatment. *BnMTP8.1* and *BnMTP8.6* were significantly induced by PEG treatment. *BnMTP9.2* and *BnMTP10.1* were slightly induced by salt stress. Under hormone treatment, we found five genes (*BnMTP9.1*, *BnMTP10.1*, *BnMTP10.2*, *BnMTP10.3,* and *BnMTP11.1*) were significantly upregulated after 3 h of ABA treatment. *BnMTP12.1* and *BnMTP12.1* were induced with GA, IAA, KT, and SL treatment for 1 h, while *BnMTP9.2* and *BnMTP10.3* were down-regulated after hormone treatments.

### 3.9. Expression of BnMTPs under Heavy Metal and Selenium Treatment

**Figure 11:**
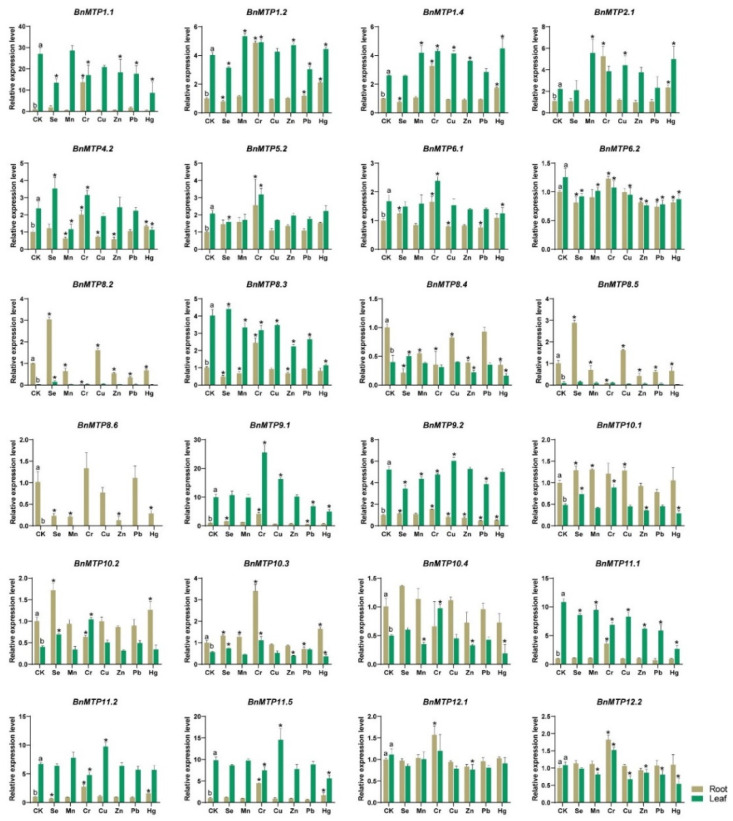
The expression level of *BnMTPs* under different heavy metal treatments. CK represents the control samples under normal growth conditions. Data represent means ± SD. Different letters (a and b) indicate significant differences between root and leaf under normal conditions. Asterisks indicate significant differences between the treatment samples and CK (*n* = 3, *p* < 0.05).

To further explore the potential biological function of *BnMTP* genes in heavy metal transportation, we analyzed the expression pattern under different metal treatments, including microelements (e.g., Zn, Cu, and Mn) and non-essential elements (e.g., Cr, Pb, and Hg). We also included selenium (Se) treatment, since rapeseed is known as an ideal crop with strong selenium enrichment ability (Figure 11). We found 24 *BnMTPs* expressed in the roots and/or leaves, and 10 *BnMTPs* with low expression in the roots and leaves under normal conditions or heavy metal treatments. Under normal conditions, 13 *BnMTPs* were more highly expressed in the leaves than in the roots, and 8 *BnMTPs* were more highly expressed in the roots than in the leaves. *BnMTP6.2*, *BnMTP12.1*, and *BnMTP12.2* were identified with similar expression in roots and leaves. Under metal treatments, the expression of 24 *BnMTPs* was significantly changed, and each *BnMTP* gene responded to one or more metal ion treatments. We summarized the fold change of *BnMTP* genes under heavy metal treatments (Table 2). In leaf, nine *BnMTP* genes (*BnMTP1.1*, *BnMTP4.2*, *BnMTP8.3*, *BnMTP8.4*, *BnMTP8.5*, *BnMTP9.1*, *BnMTP10.4*, *BnMTP11.1*, and *BnMTP12.2*) were downregulated under Hg treatment, while *BnMTP2.1* was upregulated. Mn enhanced the expression of *BnMTP2.1*, but decreased *BnMTP4.2* expression in leaf. *BnMTP9.1* and *BnMTP10.2* were upregulated by Cr, *BnMTP2.1* was upregulated by Cu, while *BnMTP8.5* was downregulated by Pb. In the root, 12 *BnMTPs* were upregulated and 3 *BnMTPs* were downregulated by Cr. Mn repressed the expression of *BnMTP8.6*, and Zn repressed the expression of *BnMTP8.4*, *BnMTP8.5*, and *BnMTP8.6*. Pb repressed the expression of *BnMTP8.2* and *BnMTP9.2*. Hg induced the expression of *BnMTP1.2*, *BnMTP2.1*, and *BnMTP11.5*, but decreased the expression of *BnMTP1.1*, *BnMTP8.4*, and *BnMTP8.6.* Moreover, Se inhibited *BnMTP1.1* expression in leaves and *BnMTP8.3*, *BnMTP8.4*, and *BnMTP8.6* expression in roots, but increased *BnMTP8.2* in roots and leaves, and *BnMTP8.5* expression in roots.

## 4. Discussion

Heavy metals such as Cu and Zn are necessary for normal plant growth, but a high concentration of essential and non-essential metals would lead to growth inhibition and toxic symptoms [12]. Plants have gradually evolved a series of cellular mechanisms to improve their tolerance to heavy metals [62]. Several ion transport families have been identified with functions in response to heavy metals, including the plasma membrane transporters such as ZIP proteins, the HMA family, YSL transporter proteins, the NRAMP family, and IRT proteins, as well as tonoplast-localized transporters such as the CDF family transporter (also called MTP) and vacuolar iron transporter family [63]. In this study, a total of 33 *BnMTP* genes were identified in the *B. napus* genome. These *BnMTP* genes were unevenly distributed on 13 chromosomes, with seven genes located on chromosome C04.

The phylogenetic analysis of MTP proteins among *B. napus*, *B. rapa*, *B. oleracea*, and *A. thaliana* indicated that the MTPs could be divided into three clusters. We found 14, 2, and 17 BnMTPs included in Zn-CDF, Fe/Zn-CDF, and Mn-CDF, respectively. Since the homologous to *AtMTP7* was absent in *B. napus* and only six groups of *MTPs* were identified in *B. napus,* we speculated that a small portion of *BnMTPs* may have undergone a gene-loss event during evolution. The protein length and intron number of *BnMTPs* varied significantly among the different groups, ranging from 151 to 769 amino acids and 0 to 12 introns. This might indicate that the BnMTPs has diverse functions. In particular, the protein size and molecular weight of BnMTP12.1 (769 amino acids and 86.67 kDa) and BnMTP12.2 (757 amino acids and 85.32 kDa) were significantly larger than other BnMTPs, indicating that they may have unique functions and specific evolutionary processes. Similar to previous reports [18], the modified CDF signature and cation_efflux domain were identified in all BnMTPs. Moreover, the zinc transporter dimer domain was observed in the BnMTPs of group 6, 8, and 9 (except BnMTP11.3 and BnMTP11.4). The ZT_dimer was reported as the dimerization region of the MTPs [64]; these BnMTPs with ZT_dimer structures might form homodimers or heterodimers to transport metal ions. The coiled-coil structure was identified in most BnMTPs of group 6, 8, and 9. Coiled-coils were involved in various processes, ranging from providing structural stiffness to the transduction of conformational changes [65,66], but whether this structure is related to the functional divergence of BnMTPs is unclear. The histidine-rich loop in MTP was considered to determine metal selectivity [67]. In the present study, typical histidine-rich regions were found in the BnMTPs of group 1 and 12. The length difference of these regions may be related to the transport ability of BnMTPs to specific metal ions. The consensus sequence HxxxD and DxxxD were differently distributed in three clusters of BnMTPs. Previous studies have shown that the different amino acid residues may be related to the functional differentiation and metal specificity of different CDF groups [18]. Interestingly, BnMTP6.1 and BnMTP6.2 did not possess any TMDs, suggesting that they might also play novel roles. Most *BnMTPs* in the same clade were identified with similar exon/intron structures, and BnMTP proteins in the same clade had similar conserved domains and motifs; this agreed with the phylogenetic tree constructed with the multi-sequence alignment. In general, the structure characteristics of BnMTPs were similar in the same group, but distinct among different groups, indicating they might have conserved yet diverse functions in *B. napus*.

Compared with the MTPs in three ancestral species, including 12 *AtMTPs* in *Arabidopsis*, 17 *BrMTPs* in *B. rapa*, and 17 *BoMTPs* in *B. oleracea*, we found that gene family expansion occurred in the *BnMTP* gene family. This might be due to the multiple polyploidization events during the evolution of *B. napus* [68]. In the present study, 66.7% (22/33) of the *BnMTPs* were derived from WGD or segmental duplication, which might be the main forces driving the expansion of the *BnMTP* gene family. Other duplication events were also found in the *BnMTP* gene family. Dispersed duplication produces two gene copies that are neither adjacent nor collinear, which is common in different plant genomes [69,70]. We identified nine *BnMTPs* derived from dispersed duplication, which were dispersed on different chromosomes. *BnMTP11.3* and *BnMTP11.4* were the only gene pair derived from proximal duplication, with a distance close to 6 kb. These results indicated that subgenomic duplication events such as dispersed and proximal duplication also play important roles in the expansion of the *BnMTP* gene family. Since *B. napus* is a young polyploid formed about 75 million years ago, the rapid genome expansion accompanied a large number of gene loss and recombination events [68]. The absence of *AtMTP6* orthologs might be caused by gene loss. Since the diploid parents (*B. rapa* and *B. oleracea*) of *B. napus* have experienced triploidization events, the number of gene family members in *B. napus* should be six times of that in *A. thaliana*. However, we found that only 45.8% (33/72) of *BnMTPs* were retained, indicating that extensive gene loss happened during *B. napus* polyploidization. After WGD or polyploidization, positive selection is important in the early stage of duplicate gene retention [71,72]. In this study, the *Ka/Ks* ratios of all duplicate *MTP* gene pairs were less than 0.5, indicating that they underwent strong purification selection after polyploidization.

*MTP* genes have been confirmed as having functions in the transport and tolerance of different heavy metals, including plant trace elements and non-essential elements [73,74], which might play important roles in improving heavy metal resistance or enrichment in plants. In *Brassica*, *B. juncea* and *B. napus* have been reported to have strong capacity in the uptake of trace elements and heavy metals [41,42]. Therefore, it is of great significance to study the functional characteristics of the *MTP* gene family in *B. napus*. *Cis*-acting regulatory elements play essential roles in gene transcription. In the present study, 323 and 154 *cis*-acting elements involved in hormone and stress response were identified. Three elements associated with stress responsiveness (ARE, MBS, and LTR) and five hormonal responsive elements (TGACG-motif, CGTCA-motif, ABRE, TGA, and TCA) are enriched in the promoters of *BnMTP* genes (Table S6), suggesting that *BnMTPs* could be regulated by multiple environmental and hormonal stimuli. To date, except for the transport and tolerance of heavy metals [73,74], there is no functional report of *MTP* genes in response to abiotic stresses and hormones. To analyze the potential function of BnMTPs in abiotic stress and hormone response, we screened the expression of *BnMTPs* in rapeseed under different treatments, including abiotic stresses such as cold, PEG, NaCl, mannitol, and hormone treatments such as ABA, GA, IAA, KT, and SL. Based on the transcriptome sequencing data, we found that multiple *BnMTPs* were regulated by hormones and abiotic stresses. Three *BnMTP* genes, *BnMTP1.4*, *BnMTP10.1*, and *BnMTP10.3*, were induced by cold, mannitol, PEG, and salt stress, while *BnMTP8.1* and *BnMTP8.6* were strongly induced by PEG treatment. In addition, several *BnMTP* genes, including *BnMTP9.1*, *BnMTP10.1*, *BnMTP10.2*, *BnMTP10.3*, and *BnMTP11.1*, were upregulated by ABA treatment, which was in accordance with the enrichment of *cis*-elements of ABA responsive elements in their promoters. Therefore, these genes might be potential targets or regulators in response to abiotic stress or hormone treatment in *B. napus*. Abiotic stress seriously affects the yield of agricultural crops around the world [75]. The species in *Brassica* are also sensitive to abiotic stresses; drought and salinity stresses greatly hinder the yield and adaptation of *Brassicas* across the world [76,77]. Therefore, it is very important to explore the stress response mechanism to improve rapeseed production under adverse environmental conditions. With the publication of the rapeseed genome sequence [68], a few studies focused on candidate genes and regulatory factors were reported to improve the stress tolerance (e.g., drought, salt tolerance) of rapeseed, including *BnPtdIns-PLC2*, *BnLAS*, *BnTTG2*, *BnALA*, *BnLEA3*, *BnVOC*, *BnaA6.RGA*, and *BnMRD107* [58,78,79,80,81,82,83]. There should be more stress-responsive genes yet to be identified in *B. napus*. In our study, several *BnMTP* genes (e.g., *BnMTP1.4*, *BnMTP8.1*, *BnMTP8.6*, *BnMTP10.1*, and *BnMTP10.3*) were identified with putative functions in the stress response, and these genes would be valuable in the genetic modification and breeding of rapeseed with stress tolerance. Phytohormones are key regulators of plant growth and development, and can also enhance plant adaptation to various abiotic stresses [84]. ABA is a phytohormone with important roles in plant responses to adverse environmental stimuli, and is involved in regulating physiological processes ranging from stomatal opening to protein storage, as well as increasing plant resistance to many abiotic stresses such as drought, salt, and cold stress [84,85]. In this study, several *BnMTPs*, such as *BnMTP9.1*, *BnMTP10.1*, and *BnMTP10.3*, were induced by both ABA and abiotic stresses, suggesting that these *BnMTP* genes might regulate plant response and tolerance to abiotic stresses via the ABA signaling pathway. miRNAs and their target genes have been reported with functions in various physiological and biochemical processes of plants under heavy metal stress, including metal uptake and transport, metal chelation, reactive oxygen species clearance, and hormone signal transduction [86,87]. A total of 12 miRNAs were identified as targeting *BnMTP* genes, and some of these miRNAs have been reported in response to different stress conditions. For instance, *miR390* and *miR156* mediated lateral root growth under salt stress, and were involved in cadmium tolerance and accumulation [88,89,90,91]. In addition, *bna-miR6031* expression was suppressed under various stress treatments [92]. Thus, the functional study of *BnMTP* genes’ response to stresses would be valuable in the future.

We also identified the specific tissue expression pattern of different *BnMTP* genes, which might be helpful to understand the potential function of *BnMTPs*. For example, *BnMTP8.1*/*8.6* and *BnMTP10.1*/*10.3*/*10.4* were highly expressed in flower and bud, whereas *BnMTP9.1* and *BnMTP11.1*/*11.5* were highly expressed in cotyledon. These *BnMTPs* might play roles in floral and cotyledon development. In addition, we found that the homologous *BnMTPs* in the same group exhibited similar tissue expression patterns, indicating that they might have similar and redundant functions, and their functional diversification might be related to the polyploidization and sequence expansion [70]. During allopolyploidization, gene duplication was usually accompanied by epigenetic-induced gene silencing, to maintain normal plant growth and development [93,94]. In *B. napus*, *BnMTP2.1*/*2.2*, *BnMTP3.1*, and *BnMTP8.2* exhibited no expression in any of the tested tissues, and this might be important to maintain the intracellular metabolic system and adaptation to environmental changes.

To investigate the potential roles of *BnMTPs* in response to heavy metal stress, we examined the *BnMTP* expression under treatment of six heavy metal ions and found the expression of several *BnMTP* genes increased or decreased significantly after Hg and Cr treatment. Hg and Cr are highly toxic to plants, with strong impacts on plant growth and crop yield [95,96]. To date, there is no report of *MTP* gene response to Hg and Cr toxicity; thus, further functional studies of these *BnMTPs* are necessary. Previous studies have shown that the expression of some *MTPs* was relatively constant at the transcription and translation levels, similar to housekeeper genes, which were not affected by the potential metal substrates [7,26,27,97]. For instance, AtMTP1 was involved in transferring excess zinc from the cytoplasm to vacuoles to maintain zinc homeostasis, but its expression level was relatively stable and was not affected by the Zn concentration [7]. In addition, the response of some *MTP* genes (e.g., *CsMTP1* in cucumber) to metal substrates might occur at the post-transcriptional level rather than the transcriptional level [97]. Recent studies have found that MTP12 could form a functional complex with MTP5 to transport Zn to the Golgi apparatus, but the expression level was not affected by an excess or deficiency of zinc [26,27]. Similarly, we found the gene expression level of *BnMTPs* in Zn-CDFs and Mn-CDFs did not change significantly (fold change > 2) in the presence of excess metals, except for *BnMTP8.6* that was downregulated by excess Mn. Therefore, future studies focusing on the expressional changes of BnMTP proteins under excessive heavy metal treatment are necessary to fully elucidate the function of *BnMTP* genes and potential BnMTP complexes.

## 5. Conclusions

In this study, 33 MTP members in *B. napus* were identified and divided into three main clusters and seven groups. The *BnMTP* gene family experienced gene expansion and loss during polyploidization, and the homologous gene of *AtMTP7* was lost in the evolutionary history. The temporospatial expression pattern and response to different heavy metal stresses suggest that BnMTPs played important roles in the growth, development, and stress response of *B. napus*, especially in heavy metal transport, detoxification, tolerance, and enrichment. In particular, the expression levels of several *BnMTPs* were significantly increased or decreased after Hg or Cr treatment, indicating that these BnMTPs may be involved in plant response to Hg or Cr. In general, these results will be helpful for the functional study of *BnMTPs* in the future.

## Figures and Tables

**Table 1:** The potential miRNA target sites in *BnMTP* genes.

Target_Acc	miRNA_Acc.	Expectation	UPE	miRNA Length	Target Position	miRNA_Aligned_Fragment	Target_Aligned_Fragment	Inhibition
*BnMTP8.1*	*bna-miR1140*	4.5	22.827	21	783–803	ACAGCCUAAACCAAUCGGAGC	UCUUGGAUUGGUUGCGGCUGU	Cleavage
*BnMTP8.1*	*bna-miR156a*	5	14.012	21	1178–1197	UGACAGAAGAGAGUGAGCACA	CCUGAACACUCUGUUCUUUCA	Cleavage
*BnMTP8.1*	*bna-miR156d*	5	14.012	20	1178–1197	UGACAGAAGAGAGUGAGCAC	CUGAACACUCUGUUCUUUCA	Cleavage
*BnMTP8.1*	*bna-miR156e*	5	14.012	20	1178–1197	UGACAGAAGAGAGUGAGCAC	CUGAACACUCUGUUCUUUCA	Cleavage
*BnMTP8.1*	*bna-miR156f*	5	14.012	20	1178–1197	UGACAGAAGAGAGUGAGCAC	CUGAACACUCUGUUCUUUCA	Cleavage
*BnMTP8.3*	*bna-miR1140*	5	18.712	21	798–818	ACAGCCUAAACCAAUCGGAGC	UCUCGGCUUGGUUGCGGCUGU	Cleavage
*BnMTP8.4*	*bna-miR1140*	5	20.181	21	798–818	ACAGCCUAAACCAAUCGGAGC	UCUCGGCUUGGUUGCGGCUGU	Cleavage
*BnMTP10.1*	*bna-miR6031*	5	21.341	24	927–950	AAGAGGUUCGGAGCGGUUUGAAGC	ACGCUCAGCCCCUCCAGACUUCUU	Cleavage
*BnMTP10.3*	*bna-miR6031*	5	16.794	24	927–950	AAGAGGUUCGGAGCGGUUUGAAGC	ACGCUCAGCCCCUCCAGACUUCUU	Cleavage
*BnMTP10.4*	*bna-miR156b*	4	17.902	21	105–125	UUGACAGAAGAUAGAGAGCAC	AAGCAUCCUAUCUUGUGUCAA	Cleavage
*BnMTP10.4*	*bna-miR156c*	4	17.902	21	105–125	UUGACAGAAGAUAGAGAGCAC	AAGCAUCCUAUCUUGUGUCAA	Cleavage
*BnMTP10.4*	*bna-miR156g*	4	17.902	21	105–125	UUGACAGAAGAUAGAGAGCAC	AAGCAUCCUAUCUUGUGUCAA	Cleavage
*BnMTP12.1*	*bna-miR156a*	5	4.093	21	297–317	UGACAGAAGAGAGUGAGCACA	CGCCUUCACUCUCUUCUCUCC	Cleavage
*BnMTP12.1*	*bna-miR156d*	5	4.093	20	298–317	UGACAGAAGAGAGUGAGCAC	GCCUUCACUCUCUUCUCUCC	Cleavage
*BnMTP12.1*	*bna-miR156e*	5	4.093	20	298–317	UGACAGAAGAGAGUGAGCAC	GCCUUCACUCUCUUCUCUCC	Cleavage
*BnMTP12.1*	*bna-miR156f*	5	4.093	20	298–317	UGACAGAAGAGAGUGAGCAC	GCCUUCACUCUCUUCUCUCC	Cleavage
*BnMTP12.1*	*bna-miR390a*	5	23.236	21	783–803	AAGCUCAGGAGGGAUAGCGCC	CCCGCUUGCUCUUUGGAGCUU	Cleavage
*BnMTP12.1*	*bna-miR390b*	5	23.236	21	783–803	AAGCUCAGGAGGGAUAGCGCC	CCCGCUUGCUCUUUGGAGCUU	Cleavage
*BnMTP12.1*	*bna-miR390c*	5	23.236	21	783–803	AAGCUCAGGAGGGAUAGCGCC	CCCGCUUGCUCUUUGGAGCUU	Cleavage
*BnMTP12.2*	*bna-miR156a*	5	5.643	21	295–314	UGACAGAAGAGAGUGAGCACA	CGCUUUCACUCUCUUCUCUCC	Cleavage
*BnMTP12.2*	*bna-miR156d*	5	5.643	20	295–314	UGACAGAAGAGAGUGAGCAC	GCUUUCACUCUCUUCUCUCC	Cleavage
*BnMTP12.2*	*bna-miR156e*	5	5.643	20	295–314	UGACAGAAGAGAGUGAGCAC	GCUUUCACUCUCUUCUCUCC	Cleavage
*BnMTP12.2*	*bna-miR156f*	5	5.643	20	295–314	UGACAGAAGAGAGUGAGCAC	GCUUUCACUCUCUUCUCUCC	Cleavage
*BnMTP12.2*	*bna-miR390a*	5	19.162	21	798–818	AAGCUCAGGAGGGAUAGCGCC	CCCGCUUGCUCUUUGGAGCUU	Cleavage
*BnMTP12.2*	*bna-miR390b*	5	19.162	21	798–818	AAGCUCAGGAGGGAUAGCGCC	CCCGCUUGCUCUUUGGAGCUU	Cleavage
*BnMTP12.2*	*bna-miR390c*	5	19.162	21	798–818	AAGCUCAGGAGGGAUAGCGCC	CCCGCUUGCUCUUUGGAGCUU	Cleavage

**Table 2:** Overview of *BnMTP* gene expression in response to different heavy metal and selenate treatment.

Gene Name	Leaf							Root						
	Se	Mn	Cr	Cu	Zn	Pb	Hg	Se	Mn	Cr	Cu	Zn	Pb	Hg
*BnMTP1.1*	-	No	No	No	No	No	--	No	No	+++	No	No	No	-
*BnMTP1.2*	No	No	No	No	No	No	No	No	No	++	No	No	No	+
*BnMTP1.4*	No	No	No	No	No	No	No	No	No	+	No	No	No	No
*BnMTP2.1*	No	+	No	+	No	No	+	No	No	++	No	No	No	+
*BnMTP4.2*	No	-	No	No	No	No	-	No	No	+	No	No	No	No
*BnMTP5.2*	No	No	No	No	No	No	No	No	No	+	No	No	No	No
*BnMTP6.1*	No	No	No	No	No	No	No	No	No	No	No	No	No	No
*BnMTP6.2*	No	No	No	No	No	No	No	No	No	No	No	No	No	No
*BnMTP8.2*	++	No	No	No	No	No	No	+	No	---	No	No	-	No
*BnMTP8.3*	No	No	No	No	No	No	-	-	No	+	No	No	No	No
*BnMTP8.4*	No	No	No	No	No	No	-	--	No	-	No	-	No	-
*BnMTP8.5*	No	No	No	No	No	-	-	+	No	---	No	-	No	No
*BnMTP8.6*	No	No	No	No	No	No	No	--	--	No	No	--	No	-
*BnMTP9.1*	No	No	+	No	No	No	-	No	No	+	No	No	No	No
*BnMTP9.2*	No	No	No	No	No	No	No	No	No	No	No	No	-	No
*BnMTP10.1*	No	No	No	No	No	No	No	No	No	No	No	No	No	No
*BnMTP10.2*	No	No	+	No	No	No	No	No	No	No	No	No	No	No
*BnMTP10.3*	No	No	No	No	No	No	No	No	No	+	No	No	No	No
*BnMTP10.4*	No	No	No	No	No	No	-	No	No	No	No	No	No	No
*BnMTP11.1*	No	No	No	No	No	No	--	No	No	++	No	No	No	No
*BnMTP11.2*	No	No	No	No	No	No	No	No	No	+	No	No	No	No
*BnMTP11.5*	No	No	No	No	No	No	No	No	No	++	No	No	No	+
*BnMTP12.1*	No	No	No	No	No	No	No	No	No	No	No	No	No	No
*BnMTP12.2*	No	No	No	No	No	No	-	No	No	No	No	No	No	No

“+” and “-” means: 2 < fold change < 4; “++” and “--” means: 4 < fold change < 8; “+++” and “---” means: fold change > 8.

## Data Availability

Not applicable.

## Supplementary Materials

The following supporting information can be downloaded at: https://www.mdpi.com/article/10.3390/genes13050761/s1. Figure S1. Multiple sequence alignment of BnMTP and AtMTP proteins, Table S1. The primers used in this study, Table S2. The information of *BnMTP* genes in *B. napus*, Table S3. The sequence and Pfam annotation of conserved motifs in BnMTP proteins, Table S4. *Ka*/*Ks* calculation of the duplicated *MTP* gene pairs. (a) *B. napus*–*B. napus*. (b) *B. napus*–*B. rapa*. (c) *B. napus*–*B. oleracea*. (d) *B. napus*–*A. thaliana*, Table S5. Duplication type of *Bn**MTP* genes, Table S6. *Cis*-acting regulatory elements identified in the promoter regions of *BnMTP* genes, Table S7. The RNA-seq data (FPKM) of *BnMTP* genes in different tissues of *B. napus*, Table S8. The expression pattern of *BnMTP* genes under abiotic stresses and hormone treatments.
