# Supplementary material for: Genome-Wide Identification and Expressional Profiling of the Metal Tolerance Protein Gene Family in Brassica napus
Source: Genes (Basel). 2022 Apr 26;13(5):761. doi: 10.3390/genes13050761 (PMC9141485; doi:10.3390/genes13050761)
Supplement: Supplementary file 1 [file genes-13-00761-s001.zip › Figure S1.pdf]

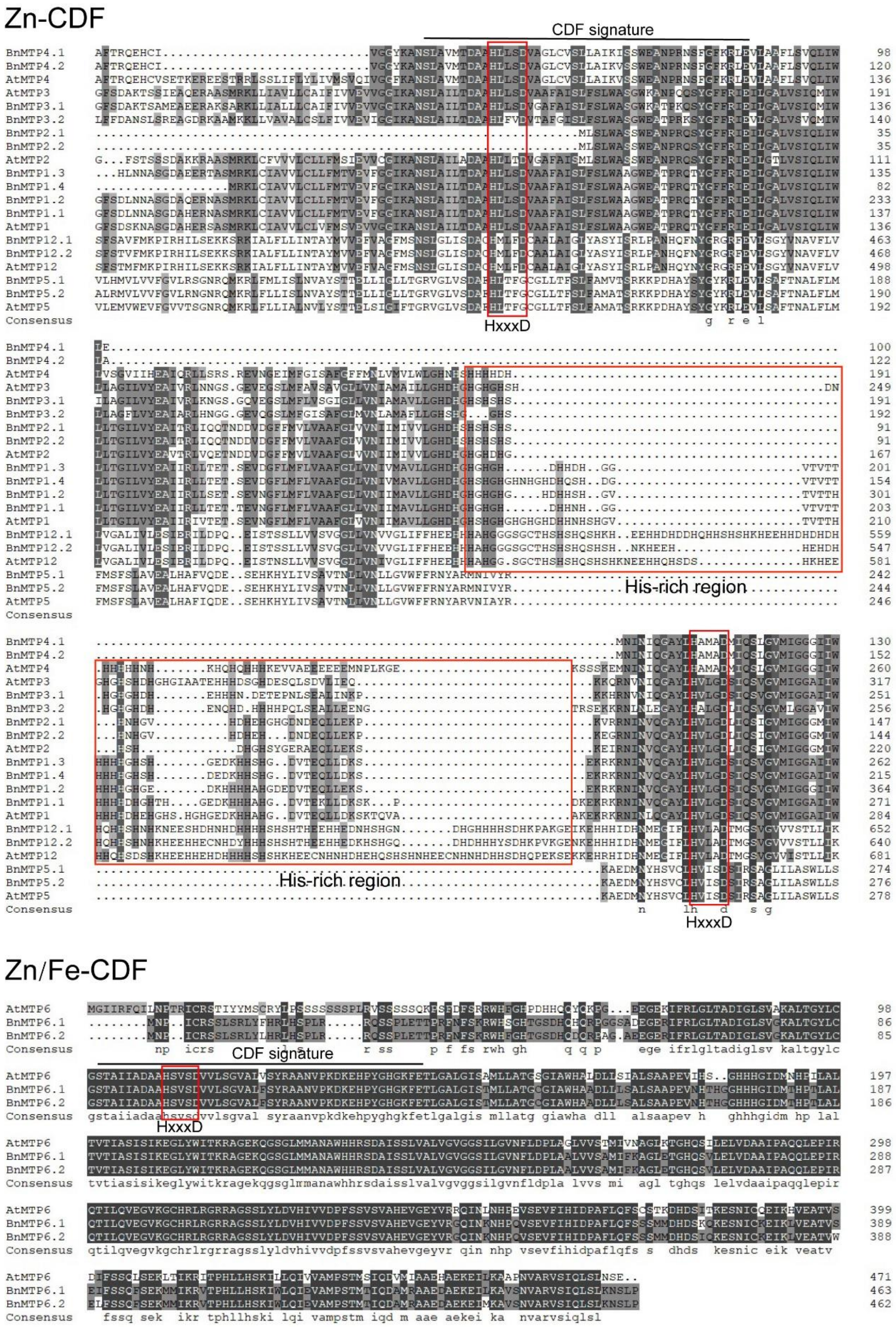
Zn-CDF
CDF signature
HxxxD
His-rich region
His-rich region
HxxxD
Zn/Fe-CDF
CDF signature
HxxxD

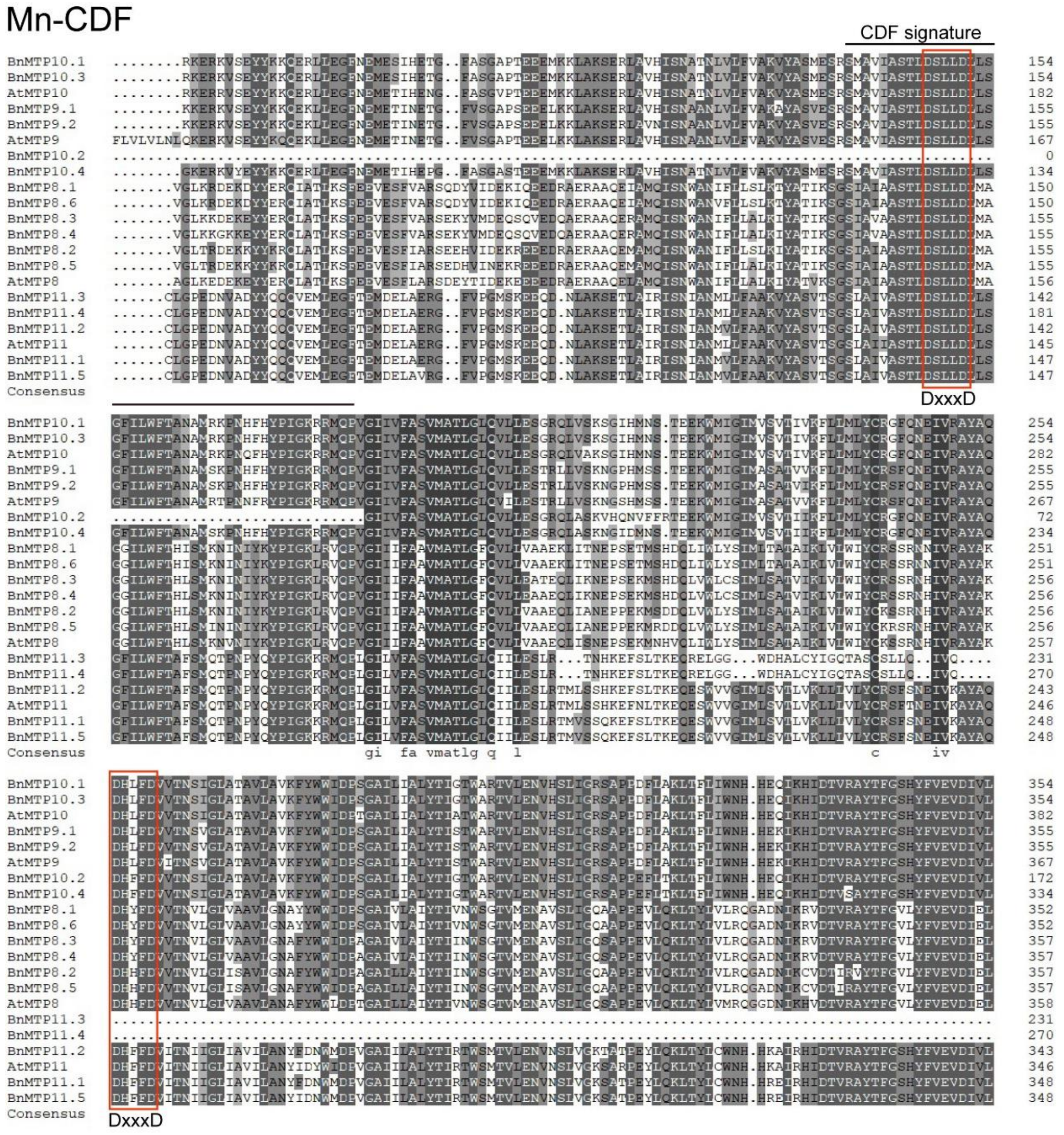

Figure S1. Multiple sequence alignment of BnMTP and AtMTP proteins. The CDF signatures were indicated with black line. The consensus sequence HxxxD (x represented any amino acid), DxxxD, and His rich region were indicated with red boxes.
